# Natural genetic variation and negative density effects in plant–nematode interactions

**DOI:** 10.1002/pei3.10133

**Published:** 2023-12-13

**Authors:** Maisara Mukhaimar, Marina Pfalz, Jacqui Shykoff, Juergen Kroymann

**Affiliations:** ^1^ Ecologie Systématique Evolution CNRS/Université Paris‐Saclay/AgroParisTech Gif‐sur‐Yvette France; ^2^ Palestinian National Agricultural Research Center – Ministry of Agriculture Jenin Palestine

**Keywords:** *Arabidopsis thaliana*, density effects, *Meloidogyne javanica*, plant–nematode interactions, root knot nematodes

## Abstract

*Arabidopsis thaliana* is a suitable host for phytoparasitic nematodes of the genus *Meloidogyne*. Successful nematode infection leads to the formation of root galls. We tested for natural genetic variation and inoculation density effects on nematode reproductive success in the interaction between *A. thaliana* and *Meloidogyne javanica*. We inoculated different Arabidopsis genotypes with two sources of nematodes at two different doses, using a mild protocol for inoculum preparation. We counted root galls and egg masses 2 months after inoculation. We obtained a high number of successful nematode infections. Infection success differed among Arabidopsis genotypes in interaction with the nematode source. Overall, infection success and reproductive success of nematodes were lower at a higher inoculum dose of nematodes. Our results indicate that natural genetic variation in both host plants and nematodes, as well as short‐ and long‐term negative density effects, shape nematode reproductive success.

## Graphical abstract

Natural genetic variation and negative nematode density effects shape the interaction between *Arabidopsis* and the root‐knot nematode *Meloidogyne javanica*.

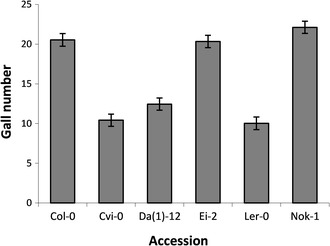

## 1 INTRODUCTION

Plant‐parasitic nematodes attack almost all crops (Sasser & Carter, 1985), causing 14% of yield loss and over US$100 billion economic loss per year (Bélair, 2005; Singh et al., 2015). Root‐knot nematodes of the genus *Meloidogyne* include more than 90 species that are responsible for about 5% of the overall yield loss. The four species *Meloidogyne javanica*, *Meloidogyne arenaria*, *Meloidogyne incognita*, and *Meloidogyne hapla* are the most widespread plant pests in the world (Eisenback & Triantaphyllou, 1991).

*Meloidogyne* species are sedentary endoparasites. First, instar (J1) larvae develop in eggs that hatch to release second instar (J2) juveniles. These migrate to the roots and penetrate them through the apex, anterior penetration zones, or small lesions, facilitating secondary infections by other parasites and pathogens. The juvenile nematodes invade the central cylinder where they establish permanent feeding sites in the root differentiation zone by inducing nuclear divisions uncoupled from cell divisions of the host cells. This process generates multinucleate giant cells. Each juvenile can trigger the development of five to seven giant cells, each of which can contain more than 100 nuclei. The parenchyma cells surrounding the giant cells undergo hyperplasia and hypertrophy, leading to the formation of root galls, which constitute the feeding sites of the nematodes. These giant cells disrupt the function of the plant vascular system. J2 juveniles molt three times before becoming adults. Adult females are sacciform and lay eggs in a gelatinous matrix. Each egg mass can contain several hundred eggs (Sharon & Spiegel, 1993).

*Arabidopsis thaliana* is a model species for genetic studies (Alonso‐Blanco et al., 2009; Alonso‐Blanco & Koornneef, 2000; Koornneef et al., 2004; Shindo et al., 2007), including those of plant defense‐related traits (Kliebenstein et al., 2001, 2002; Kroymann et al., 2003; Pfalz et al., 2007, 2009) against numerous pathogens and pests such as root‐knot nematodes (Favery et al., 2016; Noureddine et al., 2022; Pfalz et al., 2016; Silva et al., 2022; Teixeira et al., 2016). Short generation time and modest growth space requirements facilitate experimental work, and the numerous genetic and genomic resources make it possible to disentangle the genetic basis of observed variation.

Several species of plant‐parasitic nematodes complete their life cycle on *A. thaliana*, including the root‐knot nematodes *M. javanica*, *M. incognita*, and *M. arenaria*; cyst nematodes of the genus *Heterodera*; and lesion nematodes of the genus *Pratylenchus* (Sijmons et al., 1991; Wu et al., 1998), making it a particularly apt species for studying plant defense against nematodes. Previous work has uncovered natural genetic variation in Arabidopsis for root gall induction and reproductive success of female nematodes (Boiteux et al., 1999). Of note, however, was the surprisingly low success for gall formation by inoculated juvenile nematodes in this study. Boiteux et al. (1999) inoculated about 8000 juveniles and eggs and usually obtained fewer than 100 galls per plant. This modest galling success could not only result from negative density‐dependent inhibitory effects caused by the large number of nematodes in the inoculum but also from the nature of the inoculum, which consisted of a heterogeneous mixture of eggs and juveniles and hence contained an unknown number of infectious units. It is important to understand the cause of this low galling success for deciphering the genetic basis of variation in defense against these nematodes.

Here, we revisit the interaction between the apomictic root‐knot nematode *M. javanica* and Arabidopsis using an improved method of inoculum preparation that controlled the number of infectious units in the inoculum to answer the following questions: first, what is the overall success of galling by juvenile nematodes? Is the infection process very inefficient or can most juveniles produce a gall? Is there genetic variation in Arabidopsis for resistance to *M. javanica* or in *M. javanica* for infection success and reproduction on this host? Does inoculum concentration influence infection success and reproduction of *M. javanica*? To do this, we conducted experiments using different Arabidopsis genotypes and nematodes from two different sources, inoculated at different controlled concentrations.

## 2 MATERIALS AND METHODS

### 2.1 Cultivation of the root‐knot nematode *Meloidogyne javanica* on tomato

Populations of the root‐knot nematode *M. javanica* were kindly provided by Dr. Thierry Mateille (Centre de Biologie pour la Gestion des Populations, Montferrier‐sur‐Lez, France) and by Dr. Bruno Favery (Institut Sophia Agrobiotech, Université Côte d'Azur), referred to as the M‐source and the F‐source, respectively. Nematodes were maintained on the Roma variety of tomato, which is susceptible to plant parasitic nematodes. Tomato plants were grown under controlled conditions, with 14 h light at 28°C and 65% relative humidity and 10 h darkness at 19°C and 65% relative humidity in a 2:1 mixture of heat‐sterilized sand and potting soil. After the appearance of the second true leaf, tomato seedlings were transferred to fresh pots; 1 month after transfer, plants were inoculated with ca. 100 juvenile nematodes, distributed over three 2–3‐cm‐deep holes around the stem.

### 2.2 Preparation of the nematode inoculum

Root galls were typically well‐developed 1 month after inoculation. Tomato roots were washed thoroughly with tap water to remove sand and soil. Parts of the roots were placed under a stereomicroscope. Mature egg masses were removed with a scalpel (Speijer & de Waele, 1997) and collected in a 1.5‐mL Eppendorf tube containing distilled water. The suspension was poured onto a small sieve (in our case made from the neck of a plastic bottle and a mosquito net attached with a rubber band) with a mesh size of approximately 200 μm, which allowed activated J2 nematodes to pass but retained egg masses containing inactive juveniles. The sieve with the egg masses was placed in a glass Petri dish with distilled water and incubated for 1 week at 28°C in the dark. Afterward, J2 juvenile concentration was determined using a 2‐mL counting cell (Nematode Counting Slide; Chalex Corporation, Washington, USA) under a stereomicroscope (X 6). Counts were repeated three to five times to estimate nematode concentration mean and variance.

### 2.3 Inoculation of Arabidopsis with nematodes

We carried out three inoculation experiments. For the first, we chose six accessions, Col‐0, Cvi‐0, Da(1)‐12, Ei‐2, L*er*, and Nok‐1, to test for genetic variation for nematode infection success among these accessions. We inoculated each plant with an average dose of 82.5 (±2.5 SEM) J2 juvenile M‐source nematodes. These accessions vary for several important defense‐related traits, and recombinant inbred line populations from crosses between these accessions are available for mapping studies (Alonso‐Blanco et al., 1998; Lister & Dean, 1993; Pfalz et al., 2007; Simon et al., 2008). The second inoculation experiment tested the effect of the dose of infectious juvenile nematodes on infection success. We inoculated two series of ten plants of the F_3_ generation from each of four recombinant lines (A, B, C, and D) derived from a cross between two near‐isogenic lines of the Arabidopsis Da(1)‐12 x Ei‐2 population with either a high dose of J2 juveniles from the M‐source (123 ± 3.2 SEM) or a low dose (54 ± 4.2 SEM) per plant. In a third experiment, we inoculated two lines, Ei‐2 and L*er*, with nematodes from the F‐source at two different doses (42.8 ± 1.5 SEM and 143.6 ± 2.4 SEM), using 10 plants per inoculum concentration and genotype, and measured their galling and egg production.

Arabidopsis seeds were surface‐sterilized with 90% (v/v) ethanol (95% (v/v)) and 10% (v/v) sodium hypochlorite (10% (w/v)) in water. Seeds were sown in Petri dishes containing ½ Murashige and Skoog including vitamin B5 Gamborg (Duchefa Biochimie, Netherlands), 1% (w/v) sucrose (Duchefa Biochimie, Netherlands), 1% (w/v) plant agar (Duchefa Biochimie, Netherlands), and 0.1% MES (Carl ROTH, Inc., Germany), adjusted to pH 5.7 (Estelle & Somerville, 1987). After 3 days at 6°C in the dark, Petri dishes were transferred to an air‐conditioned growth chamber with 11.5 h light at 22.5°C and 60% relative humidity and 12.5 h darkness at 16°C and 70% relative humidity. Petri dishes were placed in a horizontal position for 4 days to ensure maximum light. Afterward, dishes were tilted to avoid root penetration into the nutrient medium. After 12–15 days in the growth chamber, seedlings were transferred to pots (7 × 7 × 7 cm^3^) containing heat‐sterilized sand (SACAMAT sand 02; Petruzzella, Bures‐sur‐Yvette, France) and fertilized with 25–30 mL 0.1%–0.15% (v/v) Hydrokani TMC2 (Hydro Agri France) once per week. Pots were either placed on growth trays containing 18 plants (for the first experiment) or individually on plastic saucers (for all subsequent experiments), and plants were watered every 2 days from below with 30 mL water per plant. The positions of each pot in the growth chamber or tray was re‐randomized every day. One month after transfer to sand, 1 mL of the nematode suspension of specific concentrations was pipetted into a single hole adjacent to the main stem of each plant.

### 2.4 Nematological analyses

Two months after inoculation of Arabidopsis with nematodes, plants were placed at 16°C day and night to slow nematode development to ensure that we observed only primary infections from our initial inoculum. Arabidopsis roots were washed with water and placed in a staining solution of 0.015% (w/v) phloxine B (Merck, Germany) for 15–20 min and then rinsed to remove the residual dye. Galls and egg masses were counted using a stereomicroscope, with the roots immersed in a small amount of water to avoid entanglement (Daykin & Hussey, 1985).

### 2.5 Statistical methods

Because count data may not follow a normal distribution, we analyzed count data of gall and egg numbers with generalized linear models (GLM) assuming underlying Poisson distributions using Log Link functions. Proportion data (galls per inoculated juvenile nematode and proportion of galls that bore eggs) were arcsine square root‐transformed before analysis with ANOVA. For all ANOVAs, we verified that the distribution of residuals from the model did not deviate from normality. For all analyses, “accession” or “line” and, where appropriate, “nematode dose” were considered as fixed effects. All other effects were treated as random. All analyses were carried out using JMP Version 12.0.1 (SAS).

## 3 RESULTS

**FIGURE 1:**
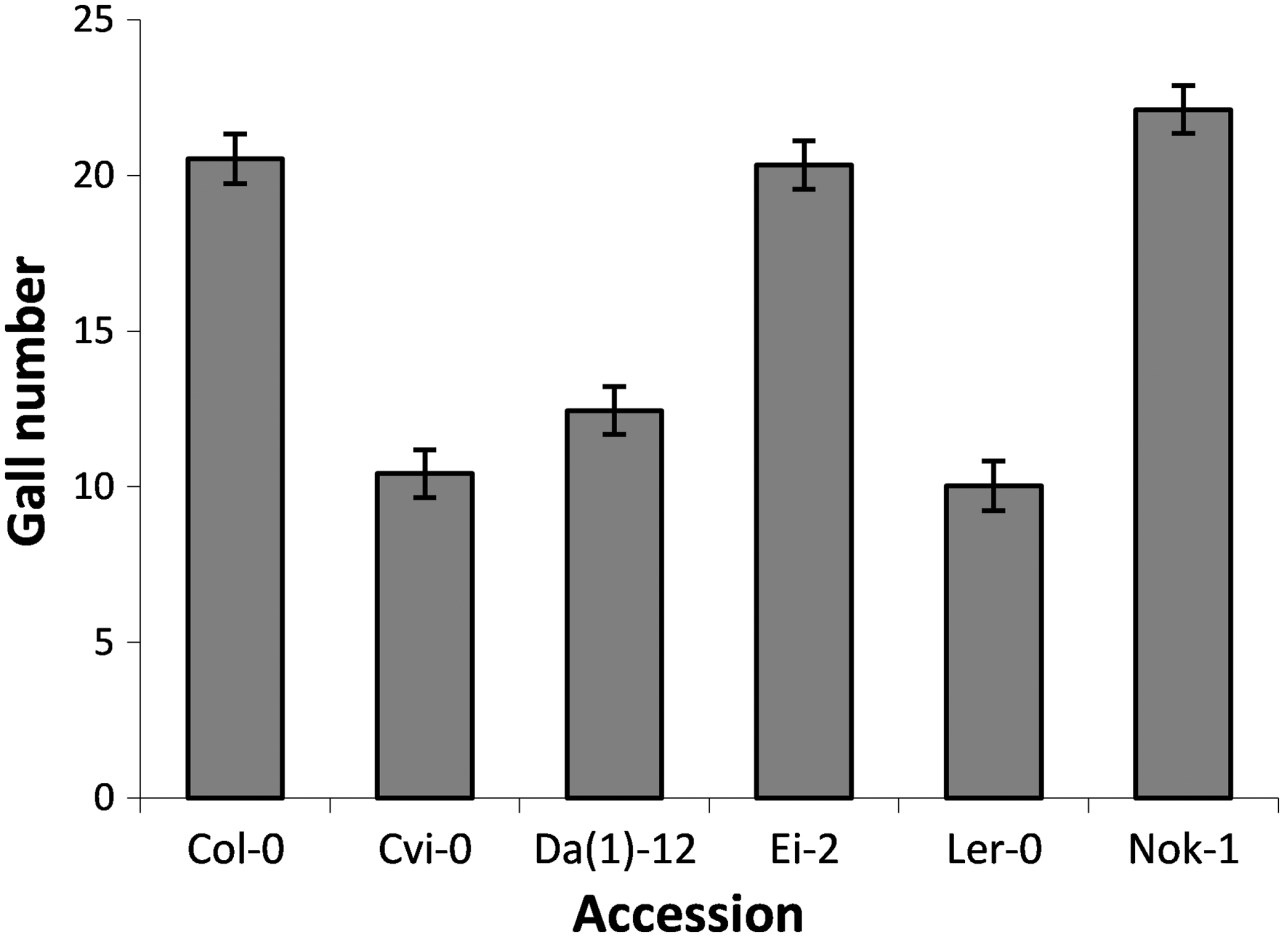
Natural genetic variation for nematode resistance among Arabidopsis accessions. Each plant was inoculated with 82.5 (±2.5 SEM) J2 nematodes and each accession had 17–18 plants distributed on six trays. Shown is the mean (±SEM) number of galls caused by *Meloidogyne javanica* on Arabidopsis roots.

We found genetic variation in resistance among six different Arabidopsis accessions, Col‐0, Cvi‐0, Da(1)‐12, Ei‐2, L*er*, and Nok‐1 (Likelihood ratio Chi‐squared = 181.28, *df* = 5, *p* < .001). Juveniles were capable of establishing feeding sites on all Arabidopsis accessions, but the number of galls varied among accessions, with Col‐0, Ei‐2, and Nok‐1 developing about twice as many galls as Cvi‐0, Da(1)‐12, and L*er*. Average infection success in this experiment varied from about 12% to 27% among accessions (Figure 1).

Next, we tested for dosage effects using four near‐isogenic lines derived from crosses between recombinant lines of the Da(1)‐12 x Ei‐2 mapping population. The majority, but not all, of the inoculated juvenile nematodes induced galls. We found an average of 36.0 galls (±1.0 SEM) for the low dose of 54 juveniles, indicating an infection success of approximately 65% (Table 1). For the high dose (about 123 juvenile nematodes per plant), we found an average of 70.6 galls (±2.0 SEM) per plant, an infection success of approximately 57%. The two‐way factorial GLM revealed significantly higher gall numbers at higher dose inoculum (Likelihood ratio Chi‐squared = 455.69, df = 1, *p* < .0001) and significant heterogeneity among lines (Likelihood ratio Chi‐squared = 9.59, df = 3, *p* = .022), but no interaction between dose and line (Likelihood ratio Chi‐squared = 2.01, df = 3, *p* > .57). Infection success per juvenile nematode was significantly greater for the low‐ than for the high‐dose treatment (*F*_1,72_ = 11.22; *p* = .0013), and we found no effect of line either as main effect or interaction (for both tests *F*_3,72_ < 2.1; *p* > .1), and the residuals from this model did not deviate from normality (*W* = 0.99, *p* > .76).

**TABLE 1:** Summary for inoculations performed with different doses of juvenile nematodes from two different sources.

Nematode source	M		F	
Number of inoculated juveniles	54 ± 4.2	123 ± 3.2	42.8 ± 1.5	143.6 ± 2.4
Galls per plant	36 ± 1.0	70.6 ± 2.0	37.8 ± 1.5	113.8 ± 5.3
Galls per juvenile (Infection success)	66.6%	57.4%	88.3%	79.2%
Egg masses per plant	28.0 ± 0.8	42.6 ± 1.6	25.3 ± 1.4	63.5 ± 3.4
Egg masses per gall (reproductive success)	77.8%	60.3%	66.9%	55.8%
Egg masses per juvenile (reproductive rate)	51.9%	34.6%	59.1%	44.2%

**FIGURE 2:**
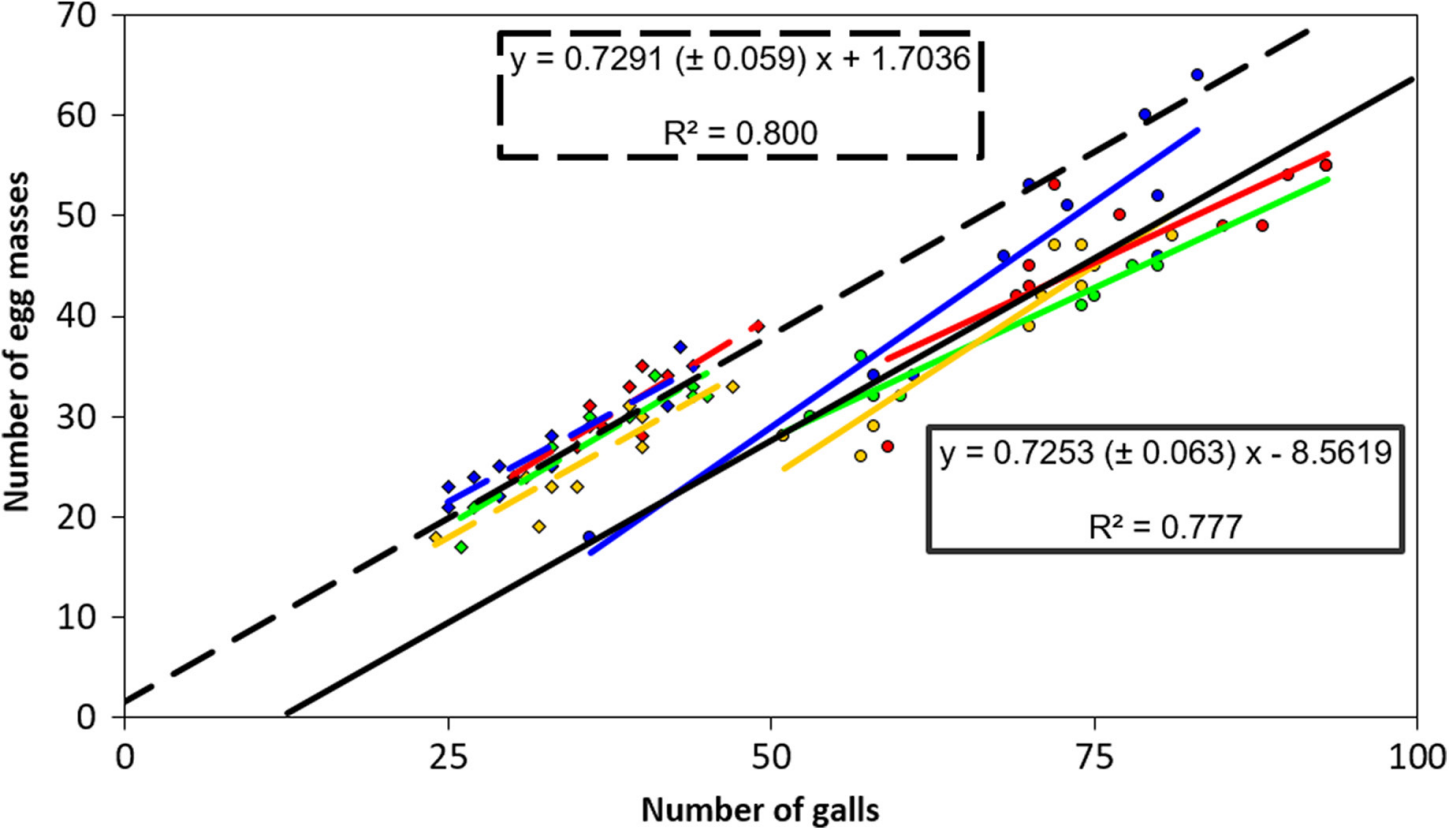
Relationship between the number of egg masses and the number of galls in recombinant lines inoculated with two different doses of juvenile nematodes. Values were measured for F_3_ plants of Arabidopsis lines A (green symbols), B (red symbols), C (yellow symbols), and D (blue symbols), which were inoculated with 54 (±4.2 SEM) nematodes (diamonds) and 123 (±3.2 SEM) nematodes (round). Per line and dose, there were 10 plants. The linear trend for each line is indicated in colors corresponding to the symbols and dotted (low dose) or solid (high dose). The trend lines in black show the linear regression on all 40 samples for the two doses, and their equations and coefficients of determination (*R* ^2^) are indicated. These trends were extrapolated to lower and higher values to facilitate comparison. Note that these trend lines are almost parallel with a slope always less than 1, but differ in intercept by inoculation dose.

Plants with more galls produced more egg masses. We analyzed egg mass number as a function of gall number, inoculum dose, and line in a full‐factorial ANCOVA GLM model. We found an effect of inoculum dose with more egg masses at higher dose (Likelihood ratio Chi‐squared = 12.60, df = 1, *p* = .0004) and a positive effect of gall number (likelihood ratio Chi‐squared = 66.12, df = 1, *p* < .0001). No other effects or interactions were significant in this analysis (all other likelihood ratio Chi‐squares <1.98, df = 1, *p* > .16), indicating that the slopes of the relationship between egg mass number and gall number did not differ between the doses. Figure 2 illustrates the relationship between egg mass number and gall number at the two doses.

**FIGURE 3:**
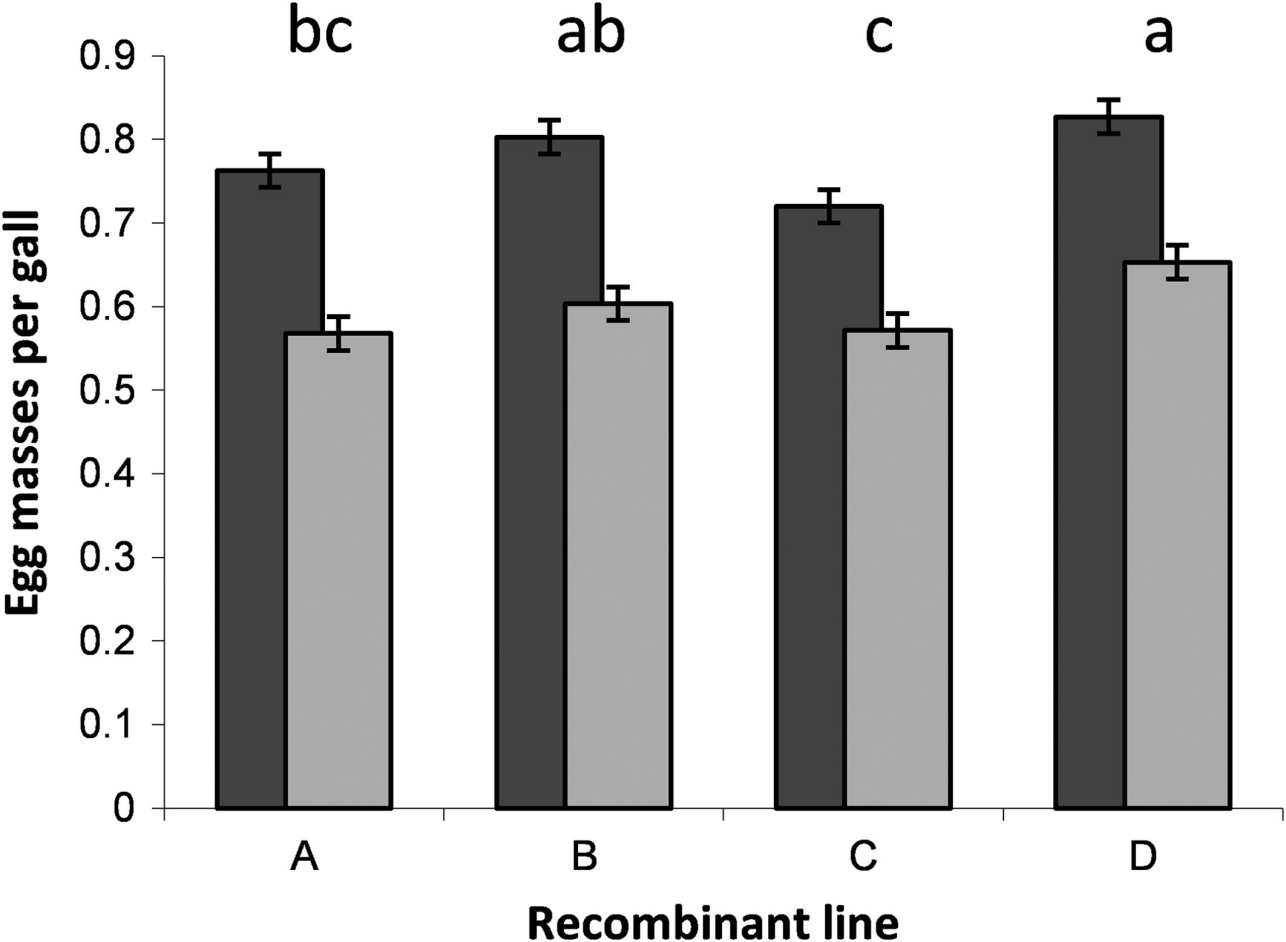
Proportion of egg masses bearing galls for four recombinant lines after inoculation with two different inoculation concentrations of juvenile nematodes. Relatively fewer galls bore egg masses at high (light gray, 123 ± 3.2 nematodes) than low inoculation dose (dark gray, 54 ± 4.2 nematodes). The proportion of galls bearing egg masses also varied among plant lines, but there was no significant interaction effect. Recombinant lines marked with the same lowercase letter did not differ at the 5% significance level, according to two‐tailed Student's *t* tests by paired combinations.

Only about 80% of galls at low inoculum concentration and about 60% at high inoculum concentration bore egg masses 60 days after inoculation (Table 1; Figures 2 and 3). On average, we found 28.0 (±0.8 SEM) and 42.6 (±1.6 SEM) egg masses per plant for the low and high inoculum concentrations, respectively. We compared the proportion of galls bearing egg masses per plant among lines, inoculation concentrations, and their interaction, after square‐root arcsine transformation. A two‐way ANOVA showed that more of the galls resulting from the low inoculum concentrations bore egg masses than those from the high inoculum concentrations (*F*_1.72_ = 155.32; *p* < .0001). Whether nematodes produced eggs or not also varied among lines (*F*_3.72_ = 9.42; *p* < .0001), but the interaction between these two factors was not significant (*F*_3.72_ = 0.81; *p* = .49), and the residuals from this model did not deviate from normality (*W* = 0.99, *p* > .57). Combining the success rates of infection on the one hand and egg production on the other, about half of the inoculated juvenile nematodes at the low and about one‐third of those at the high concentration produced eggs.

**FIGURE 4:**
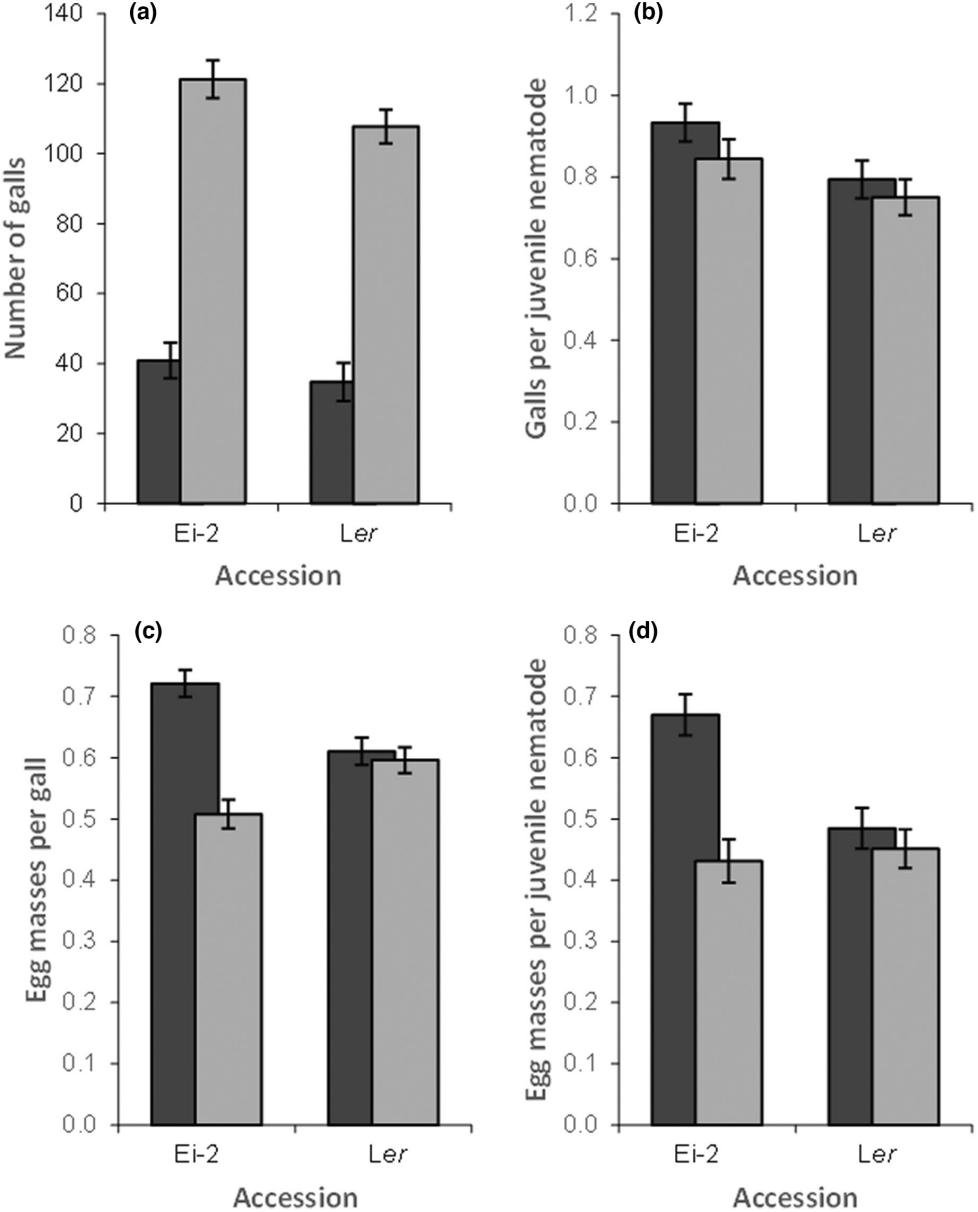
Performance of two different doses of juvenile *Meloidogyne javanica* from the F‐source on Arabidopsis accessions Ei‐2 and L*er*. Shown are the averages and standard errors of the mean of (a) galls on the plant root system, (b) galls per inoculated juvenile, (c) egg masses per gall, and (d) egg masses per inoculated juvenile. Plants were inoculated with 42.8 (±1.5 SEM; dark gray) and 143.6 (±2.4 SEM; light gray) J2 nematodes. Negative density effects are clearly visible in (c) and (d) for the Ei‐2 accession, but not for L*er*.

For the M‐source of nematodes, Ei‐2 was susceptible and L*er* more resistant (Figure 1). With the F‐source of nematodes, we found on average 78.7 (±10.5 SEM) galls on Ei‐2 and 73.2 (±9.4 SEM) galls on L*er*, with more galls at the higher dose but a similar proportion of galls per nematode at high versus low doses (Table 1; Figure 4a,b). As before, we analyzed the number of galls per plant as a function of inoculum dose, line, and the interaction between these two factors, with a two‐way ANOVA. Plants that were inoculated with the high dose of nematodes had more galls (Likelihood ratio Chi‐squared = 724.59, df = 1, *p* < .0001), and we found more galls on the roots of the Ei‐2 than on the L*er* accession (Likelihood ratio Chi‐squared = 9.95, df = 1, *p* = .0016), but there was no significant interaction between inoculum concentration and accession (likelihood ratio Chi‐squared = 0.25, df = 1, *p* = .62). Thus, though the difference in susceptibility between these two accessions was less evident for F‐source nematodes, L*er* still showed significantly higher resistance.

In this experiment, we found on average 25.3 (±1.4 SEM) egg masses for the low and 63.5 (±3.4 SEM) egg masses for the high dose of nematodes (Table 1; Figure 4c), with approximately 67% of galls bearing egg masses at low and 55% at high inoculation concentration. Thus, while the infection success was higher for the F‐source of nematodes than for the M‐source, its reproductive success was lower. The two‐way ANOVA on square‐root arcsine transformed data showed highly significant effects of dose (*F*_1,32_ = 26.79, *p* < .0001) and of the interaction between dose and accession (*F*_1,32_ = 20.39; *p* < .0001), but not of accession (*F*_1,32_ < 0.42; *p* = .52), and the residuals from this model did not deviate from normality (*W* = 0.95, *p* > .12). On average, more of the galls on plants inoculated with the lower inoculation concentration of nematodes bore egg masses. This effect was very pronounced for Ei‐2, with egg masses on 72% versus 51% of galls for low and high inoculation concentration, respectively. In contrast, successful egg mass production on the L*er* accession was indistinguishable between low and high inoculation concentration, with about 60% of galls bearing egg masses at both doses.

Successful reproduction, that is, the product of galling success and egg mass production success of juvenile nematodes in this experiment, was approximately 69% and 43% for low and high inoculation concentration, respectively, with Ei‐2 as the host and 50% and 45% for low and high inoculation concentration with L*er* as the host (Table 1; Figure 4d). Averaged across both accessions, 59% of inoculated nematodes at low dose and of 44% at high dose successfully produced egg masses.

## 4 DISCUSSION

The interaction between *M. javanica* and *A. thaliana* provides a model system for understanding variation in plant resistance to root‐knot nematodes and infection characteristics of the nematodes. In our experiments, we carefully controlled the infectious‐stage of our inoculum. By inoculating moderate numbers of infectious units (between 40 and 150 J2 nematode larvae per plant), we obtained infection successes that ranged from 12% to 88% of infectious units engendering galls. Infection success varied among plant genotypes, inoculation concentration, and experiments (Table 1; Figures 1 and 4). These were high infection rates of *A. thaliana* by *M. javanica* compared with other inoculation protocols (Boiteux et al., 1999; Sijmons et al., 1991). For example, Boiteux et al. (1999) rarely obtained more than 100 galls on *A. thaliana* whole root systems and a maximum of 215 egg masses per 100 mg of natural wet root tissue, even though they inoculated with 7900 eggs/juveniles of *Meloidogyne hapla* per plant. These values translate to success rates of less than 1% per inoculated nematode. Indeed, classic methods for inoculum preparation may generate inocula that contain many non‐infectious units. For example, (i) using sodium hypochlorite (NaOCl) to dissolve the gelatinous matrix protecting nematode egg masses (Boiteux et al., 1999; Jammes et al., 2005; Mitchum et al., 2004; Sijmons et al., 1991; Speijer & de Waele, 1997), and (ii) treatment of *Meloidogyne* juveniles and egg masses prior to inoculation with streptomycin sulfate (Speijer & de Waele, 1997) or mercuric chloride HgCl_2_ (Sijmons et al., 1991) can weaken eggshells, causing the release of premature larvae. By contrast, in their natural state, root‐knot nematode juveniles activate in the eggs first, then modify the eggshells, and hatch (Perry, 2002). Our method of inoculum preparation avoided chemical treatment and thus mimicked the natural course of hatching more closely and clearly leads to superior infection rates.

We found quantitative variation in root gall number among six Arabidopsis accessions indicating natural genetic variation in nematode resistance as has been previously observed (Boiteux et al., 1999; Sijmons et al., 1991). However, more interestingly, our inoculation protocol allowed us to examine the effect of dosage on both infection success and reproduction of these nematodes. Our inocula varied in dosage by a factor of approximately 2–3. We found lower infection success per juvenile nematode at the higher inoculum doses, and this variation was highly significant for the M‐source of nematodes. Such negative density effects are often observed for parasite–host interactions (e.g., Chandra et al., 2010). The fitness of an individual parasite is typically negatively correlated with the number of parasites that invade a host, either due to host defense responses or competition among parasites for limited resources (Ashworth et al., 1996; Ebert et al., 2000; Keymer, 1982; Luong et al., 2011; Walker et al., 2009). Such effects may, at least partially, explain the low infection success following inoculation of Arabidopsis with a very large number of nematodes (Boiteux et al., 1999).

Nematode dose in the inoculum also had a highly significant effect on reproduction of the nematodes that had successfully established. When plants were inoculated with the higher dose of nematodes, egg mass production was lower per gall measured 60 days after inoculation (Figure 3). This shows that the negative effects of density are expressed both for infection success and for reproduction. Several studies also find that reproductive success of root‐knot nematodes depends on the initial number of inoculated nematodes (Chandra et al., 2010; Charegani et al., 2012; Di Vito et al., 1986, 2004; Vovlas et al., 2005). We suggest two non‐mutually exclusive mechanisms to explain these negative density effects. Direct competition among juvenile nematodes in the inoculum may reduce their performance both for infecting the host plant and for egg production after infection. Alternatively, more juvenile nematodes trying to infect a plant could more effectively trigger plant defense systems, making the plant a poorer host for these nematodes. Indeed, *M. incognita* inoculation induces lignin biosynthesis pathway genes that could serve a priming function (Veronico et al., 2018). Distinguishing between these two mechanisms is difficult, but if plants whose defense system was completely impaired were available, one could determine their relative importance. Negative density dependence in the absence of any plant defense could only be due to direct competition among nematodes.

We carried out very similar experiments using two different nematode sources and found that infection success and reproduction on different Arabidopsis genotypes varied somewhat between the two. For example, the F‐source of nematodes was better at infecting the L*er* accession than was the M‐source (Table 1). This suggests that these two nematode sources differed for important traits associated with host use ability. Such variation for ecologically important traits is unexpected because genomic studies of *M. javanica* found little genetic variation among populations from different continents (Szitenberg et al., 2017). Nonetheless, populations of *M. javanica* regularly overcome plant resistance, so populations can vary for host breadth and exploitation ability (Ornat et al., 2001; Tzortzakakis et al., 2005) as we found here, suggesting the maintenance of genetic variation in these nematodes for ecologically and agronomically relevant traits.

We found variation in resistance to root‐knot nematodes among accessions of *A. thaliana* for which recombinant inbred line mapping populations exist. This would allow mapping of the resistance factors and, ultimately, cloning of resistance genes that could be useful for crop improvement. However, our observation of variation in the degree of resistance of the same accessions to different nematode populations of the same species suggests that resistance genes may be highly specific. Therefore, when seeking new resistance genes, it is important to test a range of different nematode populations to determine whether these resistance factors are general.

## CONFLICT OF INTEREST STATEMENT

Authors declare no conflict of interest.

## Data Availability

The manuscript contains summary data of all conducted experiments. The corresponding author will provide the original data upon request.
